# A Comparison of Self, Informant, and Researcher Rating on Mild Behavioral Impairment Checklist (MBI‐C)

**DOI:** 10.1002/alz70857_104077

**Published:** 2025-12-25

**Authors:** Tzung‐Jeng Hwang, Cho‐Hsiang Yang, Yi‐Ting Lin

**Affiliations:** ^1^ National Taiwan University Hospital, College of Medicine, National Taiwan University, Taipei, Taiwan; ^2^ National Taiwan University Hospital‐Bei Hu Branch, Taipei, Taiwan, Taiwan; ^3^ National Taiwan University Hospital, Taipei City, Taiwan

## Abstract

**Background:**

Neuropsychiatric symptoms have been considered a risk factor or prodromal state of cognitive decline and further progression into dementia. The Mild Behavioral Impairment Checklist (MBI‐C) is a newly developed instrument for assessing the presence and severity of neuropsychiatric changes. However, ratings may be susceptible to biases and discrepancies across different information sources.

**Method:**

We recruited participants who did not meet the diagnostic criteria for dementia or other major psychiatric illnesses from both family members of patients and hospital volunteers at the psychiatry department of a university medical center. All participants underwent clinical and cognitive evaluation using the CDR and MMSE and were subsequently classified as either cognitively normal or mild cognitive impairment (MCI). Then, each study participant, informant, and researcher completed the respective version of MBI‐C. The researcher's evaluation was based on information collected from the participant and close informant, so it could be considered the gold standard. The MBI criteria outlined by the International Society to Advance Alzheimer's Research and Treatment (ISTAART) was applied to identify participants with MBI. Data were analyzed to examine the differences in MBI‐C rating among the three groups.

**Result:**

A total of 123 participants with MBI (mean age of 66.8 years, 49 with pure MBI, 74 with MBI and MCI) were identified. Significant differences in 3 domain scores (decreased motivation, affective dysregulation, and social inappropriateness) were observed across the three groups. In the decreased motivation domain, both the self and researcher rating yielded significantly higher scores than the informant one (*p* < 0.001), with average scores ranging from 1.95 (self‐rating) to 1.09 (informant rating). In the affective dysregulation domain, the researcher rating scored significantly higher than the informant rating, with mean scores of 2.57 and 1.9, respectively (*p* = 0.019). However, within the social inappropriateness domain, the informant rating yielded significantly higher scores than the self‐rating, with mean scores of 0.37 and 0.13, respectively (*p* = 0.012).

**Conclusion:**

The findings suggest that the respondents' perspectives may influence MBI‐C scores. Significant differences may exist between self‐rating and informant rating in decreased motivation, affective dysregulation, and social inappropriateness domains. Clinicians should exercise caution when interpreting the results from different information sources.

